# Delayed brain and spine migration of a retained SEEG electrode fragment: An unexpected complication

**DOI:** 10.1002/epd2.70230

**Published:** 2026-03-31

**Authors:** Manel Krouma, Julia Makhalova, Gauthier Toutain, Francesca Pizzo, Stanislas Lagarde, Anne Lepine, Fabrice Bartolomei, Didier Scavarda

**Affiliations:** ^1^ Department of Pediatric Neurosurgery, La Timone Hospital Aix‐Marseille University Marseille France; ^2^ Epileptology Department, APHM Timone Hospital Marseille France; ^3^ INS, Institute of Systems Neuroscience Aix‐Marseille University, INSERM Marseille France; ^4^ Department of Pediatric Neurology, La Timone Hospital Aix‐Marseille University Marseille France

**Keywords:** child, drug‐resistant epilepsy, SEEG

## Abstract

**Background:**

Stereoelectroencephalography (SEEG) is a well‐established technique for localizing epileptogenic zones in patients with drug‐resistant epilepsy, including children. While considered safe, rare but serious complications can occur.

**Case Presentation:**

We report the case of a girl with tuberous sclerosis complex and drug‐resistant epilepsy who underwent SEEG implantation as part of pre‐surgical evaluation. Electrode placement was uneventful. However, during SEEG monitoring, the signal was lost on two electrodes, raising suspicion of fracture. During explantation, these electrodes were not retrieved, as the distal ends were not visible. The removal of the remaining electrodes proceeded without complication. Nearly 3 years later, the patient developed progressive gait instability. Imaging revealed migration of one electrode fragment into the spinal canal at the L4–L5 level, in contact with the cauda equina, and another fragment into the left temporal horn of the lateral ventricle. The spinal fragment was successfully removed surgically without complication.

**Conclusion:**

To the best of our knowledge, this is the first reported case of brain and spinal migration of a retained fragment of a SEEG electrode. This case highlights a rare but potentially significant complication of SEEG implantation and discuss the management of electrode fragments.

**Key points**

Secondary displacement of SEEG electrodes left in pace has never been reported. This case report describes such secondary displacement of a broken electrode from the frontal into lumbar dural cul de sac/. This highlights the importance of monitoring and probably removing broken electrode after SEEG.

## 1 INTRODUCTION

Stereoelectroencephalography (SEEG) has become a widely used procedure in the presurgical evaluation of drug‐resistant epilepsy (DRE) since it was first described by Talairach and Bancaud in the 1960s.^1^ By implanting multiple intracerebral electrodes with high spatial precision, SEEG provides critical data for localizing epileptogenic zones and defining their relationship with functional cortical areas. Its efficacy in guiding epilepsy surgery has been well established, and it is associated with a favorable safety profile in both adults and children.^2^, ^3^, ^4^

Despite the invasive nature of the technique, complications are rare, with a reported prevalence of approximately 1.3%.^2^ The most common adverse events include intracranial hemorrhage and infection.^2^ One uncommon but reported complication is electrode fracture during implantation, recording or explantation,^2^ which may result in the retention of a fragment within the brain.

We report a previously undocumented complication: delayed brain and spinal migration of a retained SEEG electrode fragment, ultimately necessitating surgical removal. To the best of our knowledge, this is the first such case described in the literature. This report is structured in accordance with the CARE (CAse REport) guidelines to ensure completeness and transparency.

## 2 CASE PRESENTATION

### 2.1 Medical history

**FIGURE 1:**
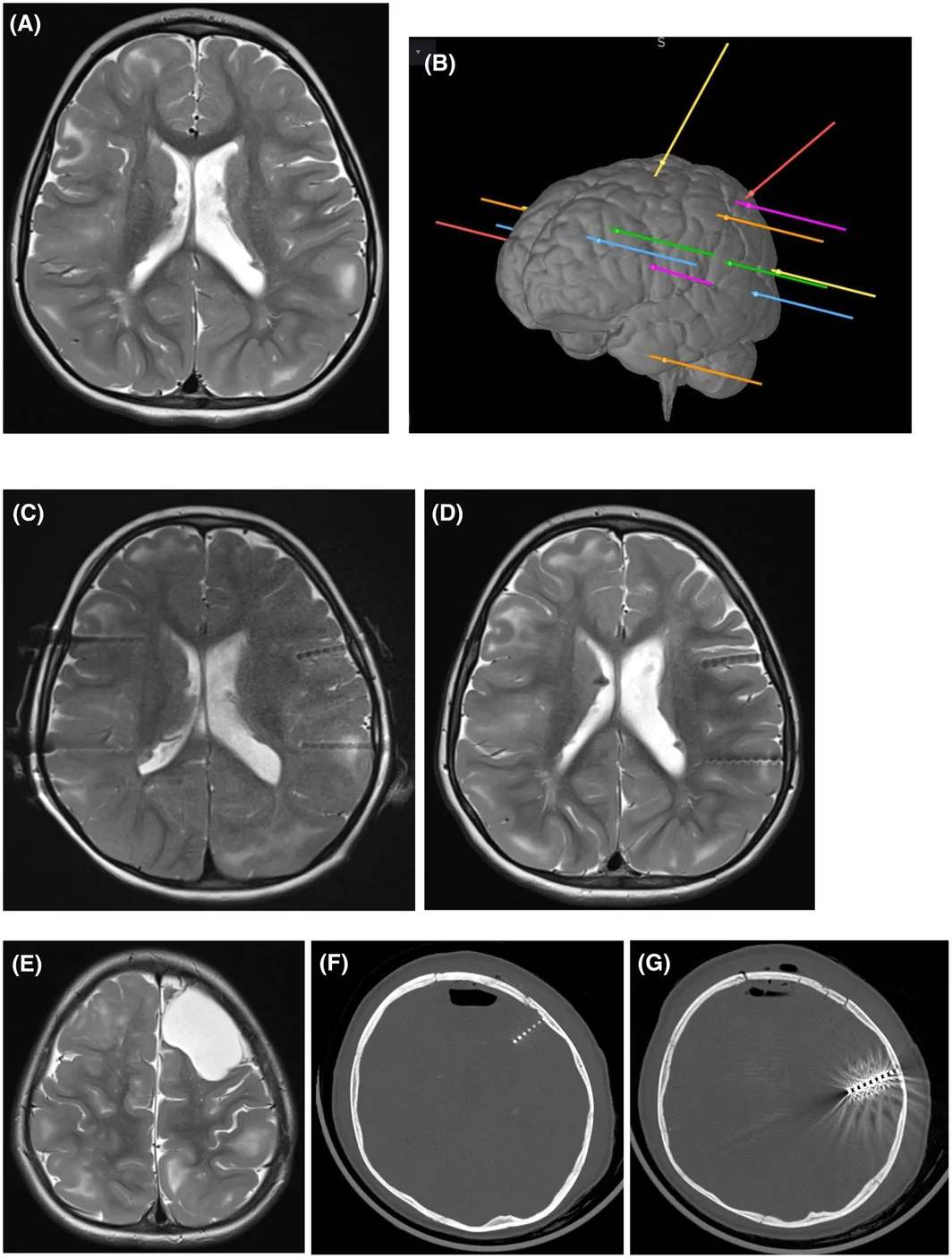
Pre‐ and post‐SEEG imaging. (A) Preoperative axial brain MRI showing multiple cortical tubers. (B) SEEG planning image illustrating the placement of the 15 electrodes. (C) Postoperative axial brain MRI showing placement of SEEG electrodes. (D) Axial brain MRI performed 2 months after SEEG implantation revealed the two fractured and retained electrode fragments. (E) Post‐cortectomy MRI following left frontal tuberectomy. (F) Axial CT showing first electrode position after frontal tuberectomy. (G) Axial CT showing second electrode position after frontal tuberectomy.

We present the case of a young girl with DRE secondary to tuberous sclerosis complex (TSC). The diagnosis was established at birth due to cardiac manifestations of supraventricular tachycardia, cardiac rhabdomyoma, and achromic spots. Genetic testing revealed a de novo heterozygous nonsense mutation p.Trp1740* in the TSC2 gene (exon 41). Initial brain MRI (Figure 1A) revealed diffuse white matter abnormalities, numerous cortical tubers in both hemispheres, subependymal giant cell nodules, and two subependymal giant cell astrocytomas (SEGAs) adjacent to the foramina of Monro.

Epileptic seizures began at the age of 3 months, presenting as infantile spasms. Clinically, the seizures manifested as clusters of extensor spasms involving all four limbs, lasting 15–20 spasms per cluster and occurring up to 10–15 times per day. Electroencephalography (EEG) showed a hypsarrhythmic pattern. Several antiseizure medications were tried, including everolimus, but none were effective.

A comprehensive Phase I presurgical evaluation was undertaken. This included long‐term video EEG monitoring, magnetoencephalography (MEG), brain MRI, and 18F‐fluorodeoxyglucose positron emission tomography (FDG‐PET). Interictal epileptiform discharges were predominantly observed over the vertex and left frontal electrodes. Based on these findings, the multidisciplinary epilepsy surgery team recommended SEEG implantation, targeting cortical tubers within the left suprasylvian region.

### 2.2 SEEG procedure and follow‐up

**FIGURE 2:**
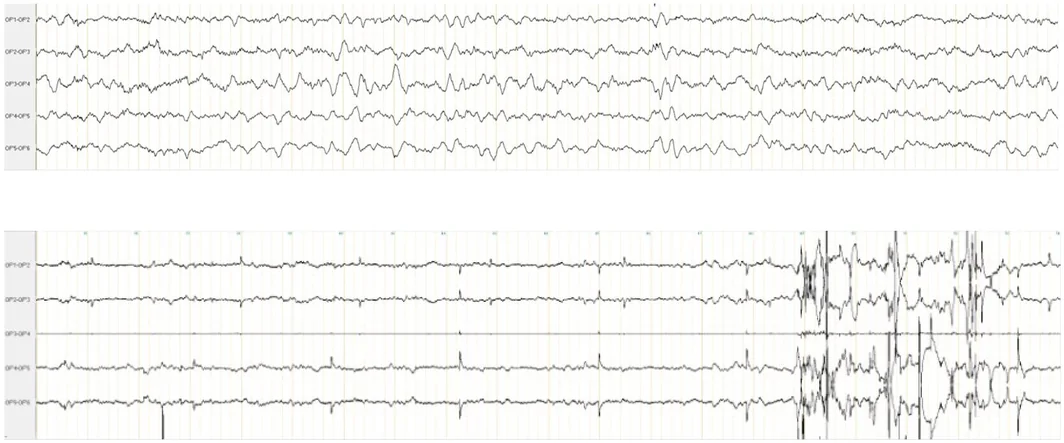
SEEG recording showing loss of signal (Electrode OF).

The patient underwent frameless, robot‐assisted SEEG with the implantation of 15 depth electrodes (Dixi, Besançon, France) using anchor bolts, 11 on the left side and 4 on the right side (Figure 1B,C). The procedure was uneventful. During the monitoring period, the patient exhibited multiple seizures. Loss of signal was noted on two electrodes during recording (3 days after the start of recording), raising suspicion of electrode fracture (Figure 2). At explantation, these two electrodes were not retrieved, as their distal tips were not visible. The remaining electrodes were removed without complication. A postoperative MRI confirmed the presence of two retained electrode fragments (Figure 1D).

SEEG analysis localized the epileptogenic zone to a left frontal cortical tuber and the patient underwent a left frontal cortectomy, removing the tuber (Figure 1E); the two electrode fragments were left in place (Figure 1F,G). Postoperative recovery was uncomplicated. Post‐surgery, seizures persisted, with incomplete control under quadritherapy.

### 2.3 Delayed complication

Nearly 3 years after SEEG implantation—the patient presented with progressive gait instability and frequent falls.

**FIGURE 3:**
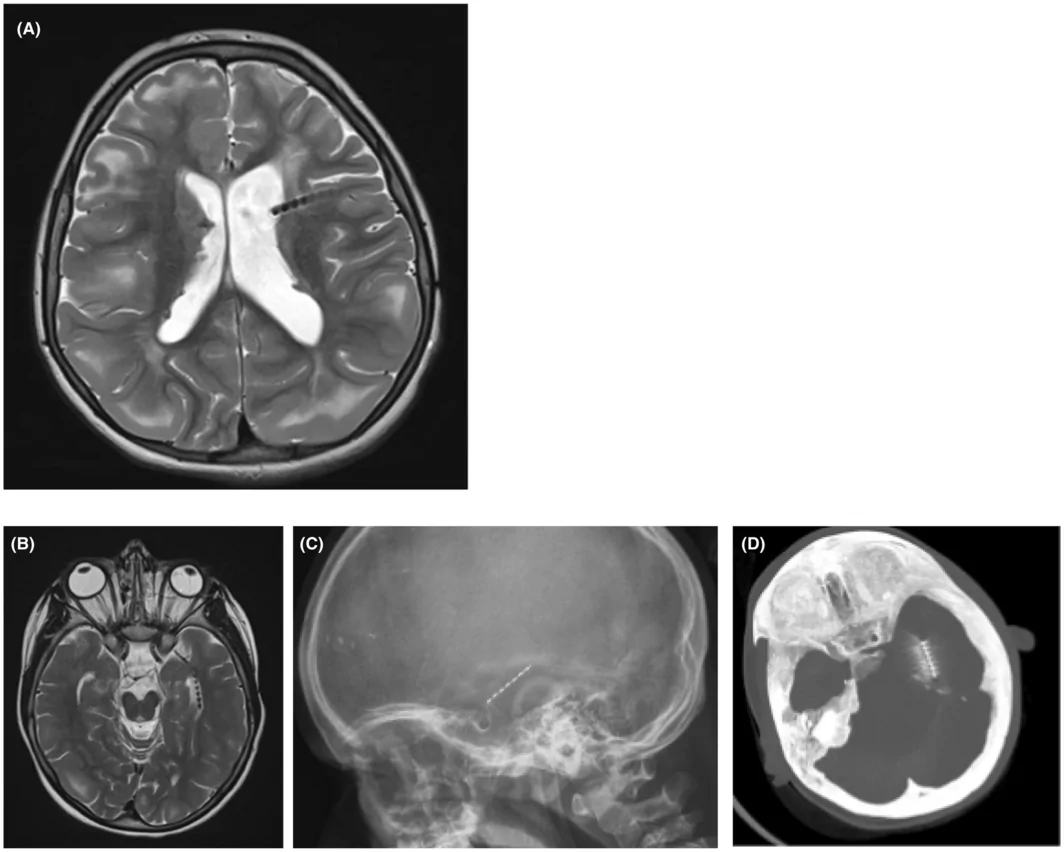
Intracranial electrode fragment migration. (A) Axial brain MRI showing initial migration of the retained electrode fragment toward the left lateral ventricle. (B) Axial brain MRI showing the position of the electrode 1 year later within the left temporal horn of the lateral ventricle. (C) Lateral skull X‐ray showing a linear, radiopaque foreign body adjacent to the left temporal squama. (D) Axial brain CT showing the position of the electrode.

Brain imaging was initially performed and showed migration of one electrode fragment into the left temporal horn of the lateral ventricle and had changed position compared to prior imaging (Figure 3). However, since two electrode fragments were known to be retained and only one was visualized intracranially, spinal imaging was performed.

**FIGURE 4:**
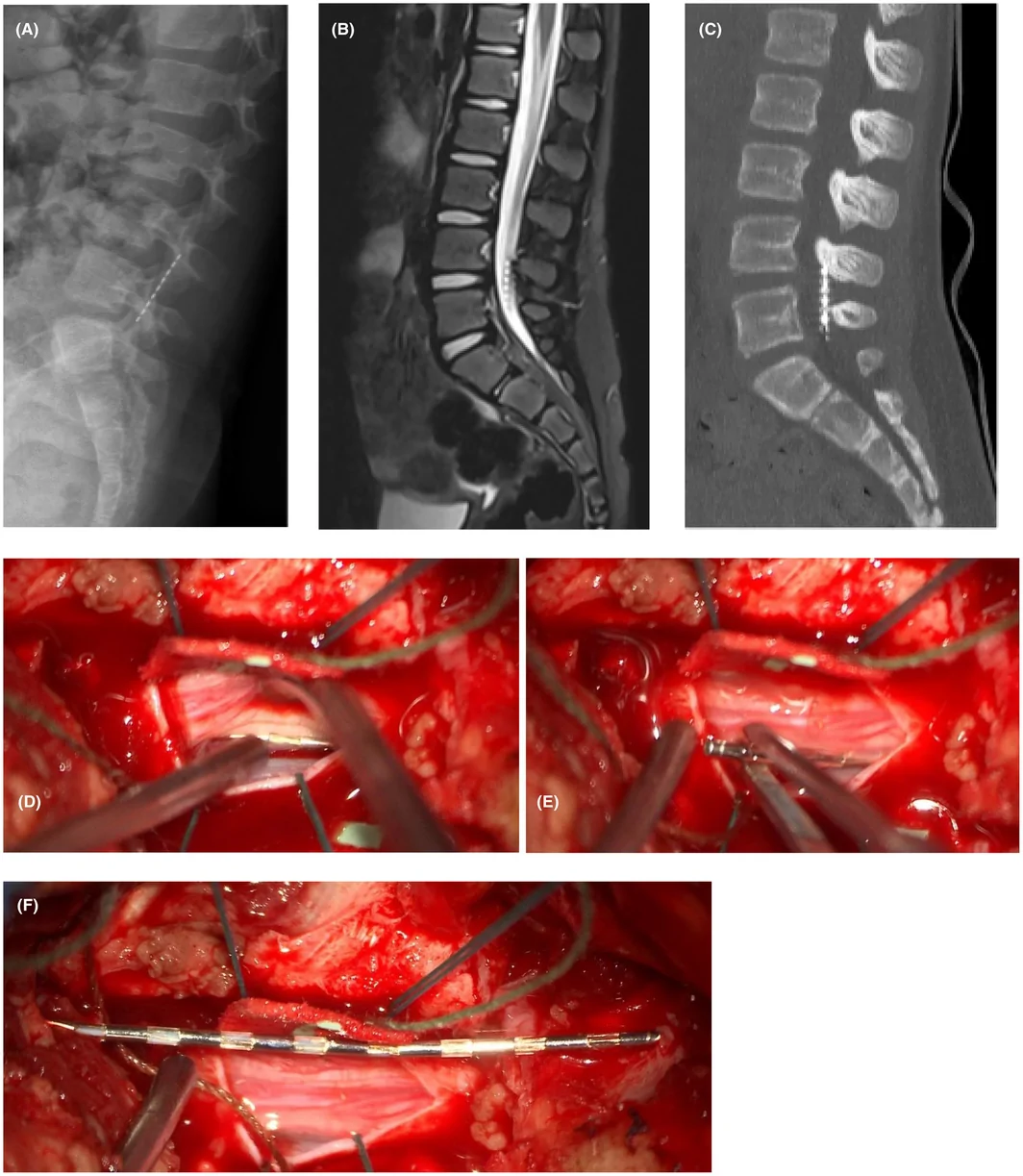
Spinal migration and surgical findings. (A) Sagittal X‐ray showing electrode position at the L4–L5 level. (B) Sagittal spine MRI showing retained SEEG electrode within the spinal canal at the L4–L5 level, in contact with the cauda equina. (C) Sagittal CT showing electrode position. (D–F) Intraoperative photograph showing the removal of the retained electrode in the spinal canal.

A lumbar spine X‐ray revealed a linear, radiopaque foreign object within the spinal canal at the L4–L5 level, consistent with migration of the second retained SEEG electrode fragment (Figure 4A). Subsequent MRI and CT confirmed the presence of this fragment at the cauda equina level (Figure 4B,C).

At the time of symptom onset, the patient was also undergoing an increase in Valproic acid dosage (132 mg/L). Partial improvement in gait was observed prior to surgery following a dose reduction. However, we could not rule out the potential contribution of the migrated electrode fragment to the patient's symptoms—by its proximity to the cauda equina—a decision was made to proceed with surgical removal. The patient underwent extraction of the migrated SEEG electrode fragment from the spinal canal. Intraoperatively, the electrode fragment was clearly visualized, not adherent to the surrounding nerves and easily removed (Figure 4D–F). Postoperatively, no neurological deficits were observed. The retained intracranial fragment located in the lateral ventricle was left in place. At the 1‐month postoperative follow‐up, the patient demonstrated significant improvement in gait and balance.

#### 2.3.1 Patient consent

Written informed consent for publication of this case and accompanying images was obtained from the patient's legal guardian.

## 3 DISCUSSION

This case describes a previously undocumented complication associated with SEEG: migration of a retained fractured electrode fragment occurring several years after implantation.

At our institution, SEEG has been performed in 173 children, with a total of 2270 electrodes implanted. Fracture occurred in 10 electrodes, corresponding to a rate of .4%. All fractures occurred during electrode explantation; no fractures were observed during the recording period.

Electrode fracture is an exceptionally rare event,^2^ and management of retained electrode fragments remains a matter of debate. Some authors recommend systematic retrieval of fractured electrode´s, whereas others recommend leaving retained fragments in situ when no immediate complication is evident, as reported cases have remained asymptomatic. To date, no complications related to retained SEEG electrode fragments have been reported.

In our center, SEEG implantation is performed using a frameless robot‐assisted technique. Electrodes are secured using anchor bolts fixed to the skull, without the need for craniotomy. In our experience, electrode fractures have occurred exclusively during explantation, typically during manipulation of the anchor bolt. When the distal electrode tip is externally visible, it is retrieved by gentle traction. In the rare cases in which the tip is not visible and the fragment migrates deeper, the fragment is left in place. Prior to the present case, no complications or migrations had been observed in our cohort. Patients were followed clinically and radiologically as part of standard long‐term epilepsy care without evidence of electrode movement.

This case represents the first instance in our experience of a migration of a retained SEEG electrode fragment. During explantation in this patient, the fractured electrode tips were not externally visible, and retrieval would have required a craniotomy. Given the absence of reported complications in our experience and in the literature, the fragments were left in situ.

During the subsequent frontal corticectomy, the question arose as to whether the retained electrode fragments should be removed. This option was not pursued for several reasons. First, no cases of delayed migration or related complications had been previously reported. Second, the cortical resection was limited to the left anterior frontal tuber located in F1 and F2, whereas the retained electrode fragment was located more posteriorly in F3 and was therefore not accessible through the craniotomy performed for tuberectomy; retrieval would have required extension of the craniotomy and likely additional cortical manipulation. Third, such an extension would have involved cortex adjacent to Broca's area and thus carried a risk of postoperative language impairment. Consequently, the risk–benefit balance favored leaving the electrode fragments in situ.

Several years later, the patient developed gait instability and imaging revealed spinal migration of one of the electrode, raising the question of whether this symptom could be related to electrode migration; however, there was no evidence of spinal canal stenosis or nerve root compression. Given that the patient's gait improved following reduction of antiseizure medication dosage, the retained electrode fragment is considered less likely to be the primary cause. Nonetheless, a contribution from mechanical irritation of the cauda equina nerve roots could not be definitively excluded. As surgical removal was considered a low‐risk procedure, the decision was made to retrieve the migrated fragment.

The patient still has another retained electrode fragment that has migrated toward the temporal horn. At present, this fragment remains asymptomatic and will be managed conservatively, with continued clinical and radiological monitoring as part of routine epilepsy follow‐up. Should further migration or signs of complication occur, surgical retrieval will be reconsidered.

The precise mechanisms underlying delayed electrode migration is not clear. Potential contributing factors include cerebrospinal fluid dynamics, gravitational forces, and cumulative body movements over time. In addition, the underlying brain pathology associated with TSC and the presence of a resection cavity may have facilitated migration.^5^ Structural abnormalities such as cortical tubers, subependymal nodules, and altered white matter architecture may reduce local tissue resistance and permit displacement of electrode fragments. Altered CSF circulation, particularly in patients with subependymal giant cell astrocytomas, may further influence both the direction and velocity of migration. Recurrent seizures and associated motor activity may also generate subtle, cumulative mechanical forces capable of displacing retained fragments over time.

This exceptionally rare case highlights a potential but previously unreported complication of SEEG and opens the discussion regarding the management of retained electrode fragments. At present, there is no consensus on systematic retrieval. Based on our experience, we recommend removal of fractured electrodes when retrieval can be performed safely and without added morbidity. However, aside from this single case, retained fragments have not been associated with adverse outcomes in our cohort. Routine additional imaging solely for the presence of an unretrievable fragment does not appear warranted, although careful long‐term clinical follow‐up remains essential.

## 4 CONCLUSION

We report the first documented case of delayed intracranial and spinal migration of retained SEEG electrode fragments. Although SEEG is considered a safe procedure with a low complication rate, this case highlights the possibility of a rare hardware‐related complication. Management in such uncommon situations are individualized, with decisions tailored to each patient's clinical context and surgical risk.

## FUNDING INFORMATION

The authors have nothing to report.

## CONFLICT OF INTEREST STATEMENT

The authors declare no conflicts of interest.

## Data Availability

The data that support the findings of this study are available on request from the corresponding author. The data are not publicly available due to privacy or ethical restrictions.
